# Supplementary material for: Heatwaves cause fluctuations in wMel Wolbachia densities and frequencies in Aedes aegypti
Source: PLoS Negl Trop Dis. 2020 Jan 23;14(1):e0007958. doi: 10.1371/journal.pntd.0007958 (PMC6977724; doi:10.1371/journal.pntd.0007958)
Supplement: S1 Table — (DOCX) [file pntd.0007958.s001.docx]

**Table S1.** General linear model for immediate effects of simulated heatwaves on *w*Mel density across life stages in *Aedes aegypti*.

| **Source** | **Type III Sum of Squares** | **df** | **Mean Square** | **F** | **P** |
| --- | --- | --- | --- | --- | --- |
| Corrected Model | 5274.292^a^ | 11 | 479.481 | 561.605 | < 0.0001 |
| Intercept | 1764.591 | 1 | 1764.591 | 2066.823 | < 0.0001 |
| Life stage | 1388.266 | 3 | 462.755 | 542.014 | < 0.0001 |
| Treatment | 2424.713 | 2 | 1212.357 | 1420.004 | < 0.0001 |
| Life stage * Treatment | 1094.039 | 6 | 182.340 | 213.570 | < 0.0001 |
| Error | 274.060 | 321 | 0.854 |  |  |
| Total | 7828.498 | 333 |  |  |  |
| Corrected Total | 5548.352 | 332 |  |  |  |
