# Supplementary material for: Heatwaves cause fluctuations in wMel Wolbachia densities and frequencies in Aedes aegypti
Source: PLoS Negl Trop Dis. 2020 Jan 23;14(1):e0007958. doi: 10.1371/journal.pntd.0007958 (PMC6977724; doi:10.1371/journal.pntd.0007958)
Supplement: Attachment — Submitted filename: Response to reviewers.docx [file pntd.0007958.s002.docx]

**Response to reviewer feedback**

**Reviewer #3: Can more information be given about the trapping methods? Particularly the prototype acoustic traps.**

We now include more details on the acoustic traps and provide references. See line 150. **L258 also refers to BGS traps, but this is not mentioned previously.**

We now state that BGS traps were used for collecting adult *Aedes aegypti* in line 149 in the methods section. In the results section (line 269) we now also refer to the acoustic traps which were not mentioned in the results.  **L287. Was the higher density in males due to an increase in the CT of the Wolbachia PCR, or a decrease in the host gene. Given males are smaller this could explain the higher ratio of Wolbachia to host.**

We usually find that host gene Cp values and Wolbachia densities are similar between the sexes. However in this case males do indeed have a higher Cp value (less DNA) for the host gene. We now include statistical tests for differences between the sexes for host and Wolbachia Cp values (lines 304-305). These tests are now also included in the methods section (lines 257-259).

**Can the heat wave related days in figure 1 be shaded or indicated by some means?**

We now indicate days where temperatures exceeded 40C with arrows and include this information in the figure legend. **Figure 3. I take it there is no significant difference in the treatments within A and B? Could this be indicated?**

There are significant differences between treatments, although they were not substantial. We now show letters indicating significant differences. Because the data were non-parametric, tests were done with Dunn’s multiple comparisons post-hoc tests, which we now mention in the figure legend.

**Reviewer #3: The discussion is rather short, and does not mention how reductions in density by temperature could affect pathogen transmission. There are studies in Anopheles system that examine the role of temperature and pathogen transmission that could be discussed. Furthermore, there appear to be quite a large variation in the density of some samples (Dec 2018). This could influence vector competence. This could be discussed in further detail bringing in the work of Gabriela Gomes which examines heterogenous effects of infections.**

We now refer to this Gomes paper and another example of temperature/Wolbachia/pathogen interactions in Anopheles at the end of the discussion. Given that there are no published examples of temperature affecting dengue blocking by Wolbachia we can only speculate at this stage. **1. Abstract and Author Summary. Although the experiments were done in North Queensland, using mosquitoes with an Australian background, I would recommend one sentence to address the fact Wolbachia deployment has been ongoing in several countries and each one is likely to face heatwaves as well. So, results observed using Australian landscape (local mosquitoes, local temperatures, local effects), those outcomes on cytoplasmic incompatibility and Wolbachia density might be seen elsewhere, showing the effects of high temperatures on maintaining long-term attributes of Wolbachia is a worth theme to investigate further.**

We have added a sentence to the end of the abstract and author summary to state that our results are relevant to releases of *w*Mel that are taking place in other countries.

**2. Line 108. The last sentence of Introduction section seems too vague. Precisely how those results on life stage-specific temperature effects on cytoplasmic incompatibility and Wolbachia density would inform Wolbachia release programs. Such affirmation requires additional clarity.**

We now specify that this can inform the choice of Wolbachia strains and timing of releases.

**3. Lines 118 and 127: The peak of temperature on Cairns was observed on late November and adult Aedes aegypti collection started on December 2018. It would add clarity if the precise dates of adult collection are provided by authors in order to see the exact temperature during field collections.**

Adults (and other life stages) were collected throughout the entire month; we now include this statement in line 148.

**4. Line 131: Considering only 4th instar larvae and adults were used for Wolbachia density, larvae from other instar and pupae were discarded?**

We included the other life stages in the presence/absence data in table 1, but excluded them from Wolbachia density analyses due to the low sample sizes. We now clarify this in lines 154-155.

**5. Line 141: What was the number of eggs collected with ovitraps? Information such as total number or average number would be important. Furthermore, what is the mean distance among ovitraps? How representative of local population the 40 ovitraps might be?**

We did not count eggs but we counted the number of larvae hatched. We now therefore include information on the % of ovitraps with *Ae. aegypti* and the median number of larvae per ovitrap in the results (lines 289-290). We do not have information on the exact location of ovitraps, but they were spread throughout several suburbs which we now list in lines 114-115.

**6. Line 155: The description of trapping with 150 ovitraps from February to March 2018 to produce baseline information should come before the later effort to check Wolbachia density.**

We have now restructured the field sampling section so that the methods for ovitrapping to establish a baseline Wolbachia infection frequency appear in the first paragraph. **7. Line 282: The fact one single ovitrap had all tested individuals negative to Wolbachia, in an area in which Wolbachia frequency is close to 100% is unexpected. Negative controls of PCR reaction worked fine? What was the distance between this negative ovitrap and the other closest one? Any local particularity worth mentioning as insecticide use by this householder, presence of pets, etc?**

The negative and positive controls worked well in all PCR runs. This trap was within the release zone and close to other traps which had a 100% infection frequency. Because we don’t have any information on the household such as insecticide/antibiotic use we can’t explain this observation, but they may be the offspring of a single uninfected female. We have previously identified full sibs emerging from the same trap (e.g. Rasic et al BMC Genomics 15:275). Wolbachia infection frequencies in field populations are almost never 100%, and since maternal transmission leakage can occur, it wasn’t surprising to see the odd uninfected sample. We now state in the results (line 297) that this result may be due to maternal transmission leakage or the loss of Wolbachia from the previous generation.

**8. Line 287 and 308: The relation between temperature and Wolbachia density seems to have different outcomes regarding mosquito sex if we go for adults reared from ovitraps, but was effectless if looking for Ae. aegypti sampled in sentinel containers. Finally, in lab assays in which the stage exposed to heatwaves varied, females had higher densities than males. Therefore, have all data in hands, each one pointing to a different relation between Wolbachia density and mosquito sex, what would be the general conclusion regarding this interaction?**

Based on these experiments and previous studies, there doesn’t seem to be any consistent sex-specific differences in Wolbachia density in response to high temperatures. The different outcomes between experiments here may reflect that Wolbachia density can change with adult age. In the lab experiments, males and females had already reproduced when they were stored for Wolbachia density, while in the ovitraps they were stored immediately after emergence. Because of these inconsistent effects we can’t state conclusively that one sex is more vulnerable to high temperatures than the other.

**9. Line 473: Authors highlighted heatwaves effects on the long-term effectiveness of Wolbachia to mitigate disease transmission, especially under replacement program strategies. However, what would be expected effects on suppression strategies? I believe it is worthwhile to add a few sentences to consider the use of Wolbachia deployment for suppression.**

We now state in the discussion (lines 480-483) that heatwaves are unlikely to affect the success of population suppression approaches because wMel in adults appears to be more resilient to temperature and that males still induce very strong cytoplasmic incompatibility even when densities are low.

**10. Figure 1: Consider highlighting (or maybe shading) the period in which adult collections were performed on the field to promote a straight comparison of field temperatures and the time in which collections were done.**

Adults were collected beginning on the 30^th^ of November and continued throughout December. We now specify this in line 148 and indicate this in Figure 1.

**11. Figure 2: I might have lost it, but why not replicate the same temperature stress in Wolbachia-uninfected individuals?**

We did include unstressed and stressed uninfected mosquitoes as controls, but not for each life stage separately. Since the controls (and previous experiments) show that heat stress does not substantially affect fertility of uninfected mosquitoes (both male and female), we did not include treatments where uninfected mosquitoes were exposed at different life stages.

**12. Figure 3. More information would be available if authors added the number of mosquitoes tested, preferentially in the legend to not pollute the graphs.**

The numbers of mosquitoes tested varied between treatments so we now indicate the ranges in the figure legend. Given that numbers varied substantially for the top two panels we would be happy to show values above each group, although we agree that this would pollute the graph.  **Reviewer #3: Should links be used in the main text?**

We included many links in the main text in our previous study published in the same journal (Ross et al. 2019 Loss of cytoplasmic incompatibility in *Wolbachia*-infected *Aedes aegypti* under field conditions), so this shouldn’t be an issue.
 **Is there any evidence from any previously published post release monitoring of lower Wolbachia loads in summer?  I am curious whether there is a threshold effect of temperature and duration that has to occur before there is an impact or whether there is a more linear relationship with rising temperatures?  Your bucket temp experiment suggests maybe 40 degrees at least is a threshold.**

No previous studies have monitored *Wolbachia* loads across seasons, only the presence or absence of *Wolbachia*. Previous experiments from our lab suggest that there is a threshold where the Wolbachia falls out rapidly, but it depends on the duration of exposure, the curve of the temperature cycle, as well as the life stage exposed. For example, even a temperature of 32C can reduce Wolbachia density if mosquitoes are exposed for long enough. **What do you think explains the different responses to heat at the different developmental stages? Interactions with immunity or rate of host cell division, etc?**

Given that heatwaves can lead to immediate reductions in *Wolbachia* density (see figure 5A) we suspect that high temperatures may simply kill the Wolbachia or prevent its replication. It could also be due to an immune response or interactions with phage and this would certainly be worth investigating in future studies. **L471. Pathogen-transmitting. Not disease-vectoring. Disease is the symptom(s) that occur in the susceptible host, not the mosquito.**

We have replaced disease with virus or pathogen when referring to transmission or blocking. **Fig 4. Examines breeding sight in sunlight or shade. What are the preferences for females to lay in these sights? One might expect females to lay in sight more suitable for development of their larvae. I presume females laid into these traps before the heat wave?**

Although mosquitoes may prefer to lay their eggs in the shade, this experiment suggests that females will still lay eggs in containers in full sunlight that reach high temperatures when provided the opportunity. We suspect that females lay their eggs in these containers during the cooler parts of the day. **Can there be some discussion as to the single ovitrap that found 100% Wolbachia negative samples. Was this in the region where Wolbachia would be expected to be found?**

This trap was within the release zone and close to other traps which had a 100% infection frequency. We now state in the results (line 297) that this result may be due to maternal transmission leakage or the loss of Wolbachia from the previous generation.

**Fig 6. Can the sample size per cage be included in the figure legend?**

We now include the number of cages per treatment and the number of females per cage in the figure legend.
